# The interplay between pain and disease activity: personal models of pain beliefs and emotional representations in children and young people with juvenile idiopathic arthritis in a UK nationwide prospective inception cohort

**DOI:** 10.1093/jpepsy/jsaf024

**Published:** 2025-04-23

**Authors:** Danielle C Mountain, Stephanie Shoop-Worrall, Lis Cordingley, CAPS PIs, G Cleary, E Baildam, L Wedderburn, J Davidson, A Chieng, F McErlane, H Foster, C Ciurtin, Y Ioannou, W Thomson, K Hyrich, Sarah Peters, Janet E McDonagh, Coziana Ciurtin, Gavin Cleary, Rebecca R Lee, Kimme Hyrich, Daniela Ghio

**Affiliations:** Centre for Epidemiology Versus Arthritis, Centre for Musculoskeletal Research, Division of Musculoskeletal and Dermatological Sciences, Faculty of Biology, Medicine and Health, University of Manchester, Manchester Academic Health Science Centre, Manchester, United Kingdom; National Institute for Health Research Biomedical Research Centre, Manchester University Hospital NHS Trust, Manchester, United Kingdom; Manchester Centre for Health Psychology, Division of Psychology and Mental Health, University of Manchester, Manchester, United Kingdom; Centre for Epidemiology Versus Arthritis, Centre for Musculoskeletal Research, Division of Musculoskeletal and Dermatological Sciences, Faculty of Biology, Medicine and Health, University of Manchester, Manchester Academic Health Science Centre, Manchester, United Kingdom; National Institute for Health Research Biomedical Research Centre, Manchester University Hospital NHS Trust, Manchester, United Kingdom; Centre for Health Informatics, Division of Informatics, Imaging and Data Sciences, Manchester Academic Health Sciences Centre, University of Manchester, Manchester, United Kingdom; Centre for Epidemiology Versus Arthritis, Centre for Musculoskeletal Research, Division of Musculoskeletal and Dermatological Sciences, Faculty of Biology, Medicine and Health, University of Manchester, Manchester Academic Health Science Centre, Manchester, United Kingdom; National Institute for Health Research Biomedical Research Centre, Manchester University Hospital NHS Trust, Manchester, United Kingdom; Manchester Centre for Health Psychology, Division of Psychology and Mental Health, University of Manchester, Manchester, United Kingdom; Centre for Epidemiology Versus Arthritis, Centre for Musculoskeletal Research, Division of Musculoskeletal and Dermatological Sciences, Faculty of Biology, Medicine and Health, University of Manchester, Manchester Academic Health Science Centre, Manchester, United Kingdom; Manchester Centre for Health Psychology, Division of Psychology and Mental Health, University of Manchester, Manchester, United Kingdom; Centre for Epidemiology Versus Arthritis, Centre for Musculoskeletal Research, Division of Musculoskeletal and Dermatological Sciences, Faculty of Biology, Medicine and Health, University of Manchester, Manchester Academic Health Science Centre, Manchester, United Kingdom; National Institute for Health Research Biomedical Research Centre, Manchester University Hospital NHS Trust, Manchester, United Kingdom; Department of Ageing, Rheumatology and Regenerative Medicine, Division of Medicine, University College of London, London, United Kingdom; Department of Rheumatology, Alder Hey Children’s Hospital, Liverpool, United Kingdom; Centre for Epidemiology Versus Arthritis, Centre for Musculoskeletal Research, Division of Musculoskeletal and Dermatological Sciences, Faculty of Biology, Medicine and Health, University of Manchester, Manchester Academic Health Science Centre, Manchester, United Kingdom; National Institute for Health Research Biomedical Research Centre, Manchester University Hospital NHS Trust, Manchester, United Kingdom; Manchester Centre for Health Psychology, Division of Psychology and Mental Health, University of Manchester, Manchester, United Kingdom; Centre for Epidemiology Versus Arthritis, Centre for Musculoskeletal Research, Division of Musculoskeletal and Dermatological Sciences, Faculty of Biology, Medicine and Health, University of Manchester, Manchester Academic Health Science Centre, Manchester, United Kingdom; National Institute for Health Research Biomedical Research Centre, Manchester University Hospital NHS Trust, Manchester, United Kingdom; Manchester Centre for Health Psychology, Division of Psychology and Mental Health, University of Manchester, Manchester, United Kingdom

**Keywords:** chronic pain, children, adolescents, young people, pediatric, juvenile idiopathic arthritis, illness perceptions

## Abstract

**Objectives:**

Juvenile idiopathic arthritis (JIA) is a group of childhood-onset inflammatory rheumatic conditions characterized by pain as one of the most common and distressing symptoms. This cross-sectional study aimed to investigate whether relationships between reported pain and disease activity in JIA affected beliefs about pain, known as “personal models.”

**Methods:**

187 out of a possible 363 participants with JIA who completed questionnaires about function and pain perception were recruited through the Childhood Arthritis Prospective Study (CAPS). A pre-selected pain score threshold and validated disease activity score cut-offs were used to assign the participants into four groups: low pain/low disease, low pain/high disease, high pain/low disease, and high pain/high disease. Multivariable linear regressions examined associations between the groups and their “personal models.”

**Results:**

Compared to participants with low pain/low disease, those with high pain/high disease and those with high pain/low disease were more likely to sense greater threat, have more negative emotional representations, and perceive less control over their pain. Participants with low pain/high disease had similar pain beliefs compared to those with low pain/low disease.

**Conclusion:**

This is the first study to compare “personal models” of pain in JIA. Children and young people who experience high pain severity regardless of disease activity perceived high pain threat, low controllability, and negative emotional representations. This highlights the importance of considering and addressing personal models of pain at diagnosis, especially those who present high levels of pain.

Juvenile idiopathic arthritis (JIA) describes a heterogeneous group of inflammatory rheumatic conditions of unknown origin and is the most common chronic rheumatic disease in children and young people (CYP) (Ravelli & Martini, 2007). JIA is a relapsing-remitting condition characterized by swelling of affected joints, stiffness, and secondary chronic musculoskeletal pain (pain in the joints, muscles, and/or bones for more than 3 months) (Prakken et al., 2011). CYP report pain as one of the most common and distressing symptoms of JIA (Kimura & Walco, 2006), describing it as unrelenting and unpredictable (Tong et al., 2012). Pain is both exacerbated by and causes changes in levels of stress and mood (Schanberg et al., 2003). Pain is influenced by interactions between biological, psychological, cognitive, and social factors (Raja et al., 2020). Best practice pain management recognizes the roles of these components (Stinson et al., 2012). Additionally, effects of JIA and pain include increased likelihood of comorbid depression (Roemer et al., 2023). Adolescents with JIA and depressive symptoms continue to experience pain and disability after a year despite reduction in disease activity (e.g., active joints) (Hanns et al., 2018).

A substantial proportion of CYP with JIA can continue to experience pain even without evidence of inflammatory disease activity (Schanberg et al., 1997; Shoop-Worrall et al., 2021). Indeed, active disease in JIA accounts for a small amount of variance of pain with little still known about how we account for the wide variations in pain experienced by CYP with JIA (Khanom et al., 2020; Lomholt et al., 2013; Packham & Hall, 2002; Stinson et al., 2012; Thastum & Herlin, 2011). In a study which investigated and identified patterns of pain over time among CYP with JIA, three trajectories were found, namely (1) CYP with consistently low pain, (2) CYP with consistently high pain, and (3) CYP with improved pain (Rashid et al., 2018). These categories are associated with disease characteristics at baseline. For example, higher initial pain intensity, a longer disease duration, and higher levels of functional disability were associated with later consistently high pain. More research is needed to understand the relationship between disease activity and pain intensity in order to inform effective pain management earlier in the disease to promote best long-term pain outcomes (Rashid et al., 2018). However, there is a tendency to address inflammation/disease first before addressing pain management, rather than addressing these symptoms in parallel (Lee et al., 2020).

We propose that the high levels of variation in responses to pain can be at least partially explained by the complex cognitive and emotional processes that individuals use to make sense of their bodily experiences (their cognitive and emotional representation of pain). These dynamic cognitive and emotional processes are based on a range of factors, including the information they are given, personal memory, feedback following activities, etc. Individuals form “Personal Models” (Hampson et al., 1990), which are a working model comprised of beliefs about their personal experiences that help make sense of health threats. For example, beliefs about the causes and consequences of their pain, its controllability, and/or curability (Leventhal et al., 2012). A systematic review has highlighted that beliefs play a crucial role in how CYP understand and manage their pain and in turn, are associated with their health outcomes, such as pain intensity and frequency (Brandelli et al., 2023). A scoping review of investigations into beliefs in pediatric pain populations found that children hold strong beliefs around impact and threat, beliefs around controllability and emotions related to these beliefs, and pain regardless of condition (Mountain et al., 2023). Beliefs about pain controllability are related to and predictive of pain intensity in healthy children (Miró et al., 2014) and physical activity as measured with accelerometry (Nørgaard et al., 2017) and pain coping strategies (Thastum et al., 2005). Despite these indications of the role pain beliefs may have in the pain experience, emphasis has been on outcomes (e.g., mental health/well-being) related to pain. Less research has focused on the types of beliefs that could be associated with and predictive of pain intensity (e.g., perceiving pain as a threat to their daily lives, negative emotional representations, and controllability over pain) (Brandelli et al., 2023). No work has investigated this relationship between beliefs, pain, and disease activity, especially for CYP who present with high pain and low disease activity. This study aims to investigate the relationship between pain and pain beliefs (perceived threat, perceived control/cure, and emotional representations) and disease activity by comparing pain beliefs across those CYP who are experiencing low pain with low disease activity, low pain with high disease activity, high pain with low disease activity, and high pain with high disease activity. It was hypothesized that CYP with high pain, regardless of their disease activity level, would perceive pain as a greater threat, have more intense emotional representations in response to pain, and perceive less control of their pain compared with those with low pain and low disease.

## Methods

### The Childhood Arthritis Prospective Study

This cross-sectional study used data from CYP recruited through the Childhood Arthritis Prospective Study (CAPS; Adib et al., 2008). CAPS is a UK multicenter prospective inception cohort of individuals diagnosed with childhood-onset inflammatory arthritis (aged ≤16 years at symptom onset). Recruitment for CAPS began in 2001 and ended in March 2018. Participants were recruited at their first pediatric rheumatology clinical consultation from seven pediatric rheumatology centers from across the United Kingdom and were followed for up to 10 years. Written informed consent from parents/carers and assent, where appropriate, from CYP were obtained. CAPS was granted approval by the Northwest Multicentre Ethics Committee (REC/02/8/104, IRAS 184042).

#### Participants

CYP from CAPS were eligible for the current study and included if they were diagnosed with JIA and aged 11+ years when they completed, or partially completed, the Pain Perception Questionnaire for Young People (PPQ-YP; Ghio et al., 2018) and had a pain severity score available at the time of completion of the PPQ-YP. To be included in the current study, CYP must have completed sections of the PPQ-YP underpinning at least one of the key outcomes (pain threat, controllability, and emotion), as detailed below.

### Data collection within the Childhood Arthritis Prospective Study

All time points within CAPS were considered for the current study. CAPS follow-up points were at initial presentation to pediatric rheumatology, annually to 5 years, and then at 7 and 10 years following this baseline visit. At each follow-up, demographic and clinical data were extracted from the clinical record by study nurses. Demographic and clinical data included: date of birth, date of symptom onset, ethnicity, ILAR (International League of Associations for Rheumatology) classification, active and limited joint counts, a 100 mm physician’s global assessment (PGA) of current disease activity, and blood biomarkers erythrocyte sedimentation rate (mm/hr) and C-reactive protein (CRP, mg/L). ILAR classification for the current study was assigned at 1 year post-inclusion in the CAPS registry. If this was missing, the closest ILAR category recorded was used, with preference given for initial classification at presentation to pediatric rheumatology. At each study time point, participants were additionally asked to complete the Childhood Health Assessment Questionnaire (CHAQ; Singh et al., 1994), incorporating a 100 mm parent or patient general evaluation (PGE) of well-being and a 100 mm pain severity score in the past week (0 = *no pain* to 100 = *very severe pain*).

### Disease activity—cJADAS

The primary exploratory variable was a combination of pain levels with disease activity status. Disease activity level was assessed using the clinical juvenile arthritis disease activity score (cJADAS-10) (McErlane et al., 2013). This score was calculated by summing PGE and PGA scores (converted from mm to cm) plus an active joint count (up to 10 joints). cJADAS scores range from 0 to 30, with higher scores indicating levels of disease activity.

### Pain/disease categories

To create the pain/disease categories for the primary exploratory variable, we measured pain levels on a 10 cm visual analogue scale to combine with disease activity status from the cJADAS. Four pain/disease categories were calculated at the point of PPQ-YP completion: “low pain plus low disease activity,” “low pain plus high disease activity,” “high pain with low disease activity,” or “high pain with high disease activity.” In this cohort study, group sizes were not manufactured to test an “optimal” ratio (see Supplementary Table S1 for power calculation). Rather, the group sizes reflect the occurrence of such outcomes in clinical practice. Pain severity scores in JIA are typically below 30 (out of 100) in CYP treated for JIA in pediatric rheumatology settings (Bromberg et al., 2014; Connelly et al., 2012; Tupper et al., 2013). Low pain was therefore defined as a pain score between 0 and 30, and high pain was defined as a score between 31 and 100. Disease activity categories were defined as per validated cJADAS cut-offs: low disease activity (scores of ≤1.5 for participants with oligoarthritis or ≤2.5 for participants with polyarthritis) and high disease activity (scores ≥1.51 for oligoarthritis or ≥2.51 for polyarthritis) (Consolaro et al., 2014).

### Pain beliefs—Pain Perception Questionnaire for Young People

The primary outcomes were three pain belief variables: beliefs about pain *threat*, *controllability* of pain, and pain *emotion* from the PPQ-YP (Ghio et al., 2018). Between 2014 and 2019, CYP completed the PPQ-YP (Ghio et al., 2018) during a routine clinical appointment. The questionnaire was provided to new recruits to the study and existing CAPS participants aged 11+ years. The PPQ-YP is a questionnaire developed from the Illness Perception Questionnaire (Moss-Morris et al., 2002) to explore CYP’s beliefs about pain. Thirty-two items assess cognitive representations: *consequences* (i.e., impact on everyday life; school, relationships, activities), pain *recurrence* (i.e., the recurring nature of pain), pain *predictability*, pain *coherence* (i.e., their overall understanding of pain), and *controllability/curability* (i.e., personal control or treatment control). Fourteen items assess *emotional representations* (i.e., how much and how often responsive emotions [anger, frustration, feeling down, or sad] and anticipatory emotions [anxiety, fear] are experienced). The PPQ-YP has demonstrated acceptability for use with young people with JIA (Ghio et al., 2018).

The three outcome variables used for this study were calculated by summing the scores of the items for each relevant pain belief construct (*threat*: consequences, recurrence, predictability; *controllability*: control, coherence; and *emotion*: anticipatory emotional representations, responsive emotional representations). The grouping was informed by work from Hagger and Orbell (2022) and resulted in three pain belief variables: pain *threat*: score range 0–170, *controllability* of pain: score range 0–130, and pain *emotion*: score range 0–140 (Table 1).

**Table 1.** Pain perception scores and missing data for each pain/disease group.

	Whole group (*n* = 363)		Low pain, low disease (*n* = 59)	Low pain, high disease (*n* = 44)	High pain, low disease (*n* = 14)	High pain, high disease (*n* = 70)	*p*
	Data available, *n* (%)	*Mdn* (IQR)	*Mdn* (IQR)	*Mdn* (IQR)	*Mdn* (IQR)	*Mdn* (IQR)	
**Cognitive representations**							
Threat (0–170) Higher scores indicate greater perceived threat of pain)	318 (87.60%)	74.90 (52.50, 96.35)	58.30 (40.00, 76.28)	77.45 (54.38, 90.93)	92.00 (74.90, 105.96)	95.00 (75.7, 109.10)	.019
Control (0–130) Higher scores indicate greater control of pain	328 (90.40%)	77.50 (63.68, 92.15)	87.65 (77.95, 101.83)	85.10 (73.10, 100.15)	69.30 (62.38, 85.98)	71.30 (54.35, 84.05)	<.001
**Emotional representations** (0–140) Higher scores indicate a greater reflection of anticipatory and responsive emotions in response to pain	323 (89.0%)	41.70 (20.40, 68.20)	31.70 (18.65, 59.50)	42.80 (15.50, 70.20)	41.95 (30.10, 63.13)	54.00 (32.50, 84.65)	<.001

*Note.* IQR = interquartile range.

### Data analysis

The current cross-sectional analysis used data from the time point of participants’ first completion of the PPQ-YP. Statistical analysis was conducted using SPSS 25.0 (IBMCorp, 2017) and Stata 14.0 (StataCorp, 2015). Descriptive statistics summarized demographic information and pain belief scores across the whole study population and within the four pain/disease status groups using median and interquartile range or frequency and percentages for a subgroup of participants with disease activity and pain scores collected at the same time point as the PPQ-YP questionnaire. Significant differences in demographic and clinical characteristics and pain belief scores between the four groups were explored using Kruskal–Wallis tests for continuous variables and chi-square tests for categorical variables. Spearman’s correlations examined preliminary relationships between the three pain belief variables with pain severity scores and cJADAS-10 scores, respectively. Three separate multivariable linear regressions were conducted to examine the association between pain/disease status and (1) pain threat, (2) pain controllability, and (3) pain emotion with the four groups of pain and disease activity. Regression models controlled for age at PPQ-YP completion, gender, disease duration to diagnosis (months), and number of years following diagnosis to PPQ-YP completion.

### Missing data

Analyses were initially completed using complete case analysis. Participants were included in the complete case analysis if their cJADAS score surpassed the “high” threshold despite having missing disease data, since the presence of additional data would not change this status. Participants with missing data and cJADAS scores not surpassing the “high” threshold were not included in the analysis, since it was not possible to determine whether their scores would be “low” or “high.” Multiple imputation using chained equations was then used over 20 iterations to impute missing disease data and demographic variables for those participants with complete ILAR and PPQ-YP data. Regression modeling was then repeated using imputed data.

## Results

### Study population

A total of 363 CYP had been recruited to the CAPS cohort by 2019 and had completed the PPQ-YP at least once (Table 2). Sixty-eight percent of the cohort were female with a median age of 15.1 years (interquartile range [IQR] 12.6, 17.5). The median age at diagnosis was 8.2 years (IQR 4.8, 12.0 years), and the median time since diagnosis was 5.0 years (IQR 2.0, 10.00) (Table 2). There were no significant differences in gender, age, symptom duration to diagnosis, ethnicity, disease activity, well-being or pain at diagnosis between those included in, and excluded from, the current study (see Supplementary Table S1). Those included had marginally higher CHAQ scores at diagnosis (*Mdn* = 0.625) than those excluded (*Mdn* = 0.750, *p* = .02), and oligoarthritis was less represented in those included (included: 41%, excluded: 51%), with RF-negative polyarthritis more represented than in those excluded (included: 28%, excluded: 21%, *p* = .002).

**Table 2.** Participant characteristics.

	Whole group (*n* = 363)		Low pain/low disease (*n* = 59)	Low pain/high disease (*n* = 44)	High pain/low disease (*n* = 14)	High pain/high disease (*n* = 70)	*p*
Characteristic	Data available, *n* (%)	*Mdn* (IQR) or *n* (%)	*Mdn* (IQR) or *n* (%)	*Mdn* (IQR) or *n* (%)	*Mdn* (IQR) or *n* (%)	*Mdn* (IQR) or *n* (%)	
Female sex	276 (76.00%)	188 (51.80%)	33 (55.93%)	24 (54.54%)	10 (71.43%)	31 (44.29%)	.110
Age, years	232 (63.90%)	15.10 (12.61, 17.48)	13.41 (12.23, 15.87)	15.06 (12.36, 16.21)	13.30 (12.37, 15.42)	14.72 (12.65, 18.56)	.160
White race or ethnicity	331 (89.50%)	299 (92.2%)	53 (89.83%)	35 (79.55%)	13 (92.86%)	57 (81.43%)	.521
Symptom duration to diagnosis (months)	187 (50.54%)	4.50 (2.30, 8.18)	3.84 (2.14, 7.69)	4.00 (2.30, 6.08)	6.77 (5.35, 8.28)	6.64 (1.97, 9.46)	.424
Years following diagnosis	322 (88.70%)	5.00 (2.00, 10.00)	7.00 (4.00, 10.00)	5.00 (1.00, 10.00)	7.00 (4.00, 10.00)	3.00 (0.00, 10.00)	.001
**ILAR category 1 year**	343 (92.70%)						
Systemic		20 (5.80%)	6 (10.20%)	4 (9.10%)	0 (0.00%)	1 (1.40%)	.264
Oligoarthritis							
Persistent		115 (33.50%)	19 (32.20%)	14 (31.80%)	6 (42.90%)	20 (28.60%)	
Extended		25 (7.30%)	2 (3.40%)	3 (6.80%)	0 (0.00%)	8 (11.40%)	
RF-negative polyarticular		95 (27.70%)	15 (25.40%)	12 (27.30%)	6 (42.90%)	20 (28.60%)	
RF-positive polyarticular		17 (5.00%)	4 (6.80%)	0 (0.00%)	2 (14.30%)	6 (8.60%)	
Enthesitis-related		23 (6.70%)	3 (5.10%)	3 (6.80%)	0 (0.00%)	8 (11.40%)	
Psoriatic		28 (8.20%)	6 (10.20%)	5 (11.40%)	0 (0.00%)	6 (8.60%)	
Undifferentiated		20 (5.80%)	4 (6.80%)	3 (6.80%)	0 (0.00%)	1 (1.40%)	
**cJADAS-10**	170 (45.90%)	2.00 (0.00, 8.00)	0.00 (0.00, 0.40)	5.00 (3.00, 9.20)	0.50 (0.00, 1.00)	9.50 (6.45, 14.25)	<.001
Active joint count	231 (63.60%)	0.00 (0.00, 1.00)	0.00 (0.00, 0.00)	1.00 (0.00, 2.00)	0.00 (0.00, 0.00)	1.00 (0.00, 5.00)	<.001
PGA, 0–100 mm	179 (49.30%)	4.00 (0.00, 20.00)	0.00 (0.00, 0.00)	20.00 (10.00, 31.00)	0.00 (0.00, 0.00)	20.50 (10.50, 40.00)	<.001
PGE, 0–100 mm	233 (64.20%)	10.00 (0.00, 39.50)	0.00 (0.00, 1.00)	20.00 (4.00, 50.00)	0.50 (0.00, 5.00)	49.00 (31.00, 64.00)	<.001
**Other disease-related characteristics**							
Limited joint count	232 (63.90%)	0.00 (0.00, 2.00)	0.00 (0.00, 0.00)	1.00 (0.00, 3.00)	0.00 (0.00, 0.00)	1.00 (0.00, 5.00)	<.001
ESR, mm/hr	167 (45.10%)	8.00 (4.00, 15.00)	6.00 (3.00, 10.00)	9.50 (2.00, 16.00)	6.00 (1.50, 11.50)	9.00 (5.00, 26.00)	.090
CHAQ, 0–3	317 (87.30%)	0.13 (0.00, 0.75)	0.00 (0.00, 0.00)	0.00 (0.00, 0.13)	0.44 (0.25, 1.38)	0.88 (0.38, 1.63)	<.001
Pain severity VAS, 0–100 mm	334 (92.00%)	19.00 (1.00, 50.00)	1.00 (0.00, 7.00)	6.00 (0.25, 19.00)	58.50 (43.00, 76.50)	59.50 (48.00, 71.00)	<.001

*Note.* IQR = interquartile range; CHAQ = Childhood Health Assessment Questionnaire; cJADAS = Clinical Juvenile Arthritis Disease Activity Score; ESR = erythrocyte sedimentation rate; ILAR = International League of Associations for Rheumatology; PGA = physician global assessment of disease activity; PGE = parental/patient global evaluation of disease activity; RF = rheumatoid factor; VAS = Visual Analogue Scale.

### Pain/disease categories

One hundred and eighty-seven participants had data available for their cJADAS and pain severity scores at the time point of a completed PPQ-YP questionnaire data collection, which allowed them to be grouped into categories of high pain/high disease (*n* = 70), high pain/low disease (*n* = 14), low pain/high disease (*n* = 44) and low pain/low disease (*n* = 59) (Table 2).

CYP in the groups with low disease had significantly longer disease duration from diagnosis to PPQ-YP completion (median (IQR), years: low pain/low disease: 7 (4, 10); high pain/low disease: 7 (4, 10); low pain/high disease: 5 (1, 10); high pain/high disease: 3 (0, 10), *p* < .001). However, there were no other demographic differences, nor a significant difference in ILAR subgroups between groups (*p* = .264). Groups with low pain had lower median CHAQ scores (median (IQR): low pain/low disease: 0.0 (0.0, 0.0); low pain/high disease: 0.0 (0.0, 0.1), with higher CHAQ scores for those with high pain/low disease (*Mdn* = 0.4, IQR 0.3, 1.4) and highest for those with high pain/high disease (*Mdn* = 0.9, IQR 0.4, 1.6) (*p* < .001) (Table 2).

#### Correlations between pain severity, disease, and pain perceptions

Table 3 presents correlations between pain severity, cJADAS, and the three PPQ-YP pain perception scores pertaining to threat, control, and emotion. Pain severity and cJADAS were positively and significantly correlated with perceptions of threat and emotion. Pain severity and cJADAS negatively and significantly correlated with perceptions of controllability.

**Table 3.** Spearman correlations between pain severity, cJADAS, and pain perceptions.

	1	2	3	4	5
1. Pain severity	–	.506^**^	.572^**^	−.464^**^	.328^**^
2. cJADAS	.506^**^	–	.336^**^	−.221*	.147
3. Threat	.572^**^	.336^**^	–	−.341^**^	.678^**^
4. Control	−.464^**^	−.221*	−.341^**^	–	−.241^**^
5. Emotion	.328^**^	.147	.678^**^	−.241^**^	–

*Note.* cJADAS = Clinical Juvenile Arthritis Disease Activity Score.

* *p* < .01, two-tailed. **p < .001, two-tailed.

#### Associations between pain severity and disease grouping with pain perceptions

Participants with high pain/high disease were significantly more likely to perceive greater threat (*p* < .001) and emotional reactions to their pain (*p* < .001) and less control of their pain (*p* < .001) than participants with low pain/low disease. Participants with high pain/low disease (*p* < .001) were significantly more likely to perceive greater threat and less control of their pain (*p* = .007) than participants with low pain/low disease. There were no significant differences in any pain beliefs for participants with low pain/high disease compared to those with low pain/low disease. The results of the complete case analysis were similar to the multiple imputation analysis (Table 4), and there was no difference when ILAR subtype was added as a confounder to the regression model (=data not shown).

**Table 4.** Associations between pain/disease status and pain threat, controllability and emotion assessed via multivariable linear regression in complete case and imputed analyses.

	CCA regression			MI regression		
Predictor groups	Coefficients	95% CI	*p*	Coefficients	95% CI	*p*
**Threat**						
Low pain/low disease	Reference	Reference	Reference	Reference	Reference	Reference
Low pain/high disease	10.11	−4.35, 24.57	.170	7.62	−3.49, 18.72	.176
High pain/low disease	34.23	15.81, 52.64	<.001	31.48	16.66, 46.29	<.001
High pain/high disease	37.99	23.61, 52.38	<.001	31.37	22.09, 40.65	<.001
**Control**						
Low pain/low disease	Reference	Reference	Reference	Reference	Reference	Reference
Low pain/high disease	4.41	−5.48, 14.30	.382	−1.88	−9.43, 5.67	.410
High pain/low disease	−13.20	−25.64, −0.75	.038	−14.42	−24.88, −3.96	.007
High pain/high disease	−20.25	−29.95, −10.56	<.001	−17.37	−2.13, −10.61	<.001
**Emotion**						
Low pain/low disease	Reference	Reference	Reference	Reference	Reference	Reference
Low pain/high disease	14.19	−3.37, 31.76	.113	7.17	−5.13, 19.46	.250
High pain/low disease	6.12	−16.12, 28.36	.590	16.18	−0.47, 32.82	.057
High pain/high disease	26.15	9.02, 43.28	.003	22.55	11.83, 33.27	<.001

*Note.* Higher scores on pain belief variables indicate greater perceived threat of pain, greater control of pain and greater emotional response to pain. All analyses adjusted for age, gender, years following diagnosis and symptom duration between onset and first pediatric rheumatology appointment. CCA = complete case analysis; MI = multiple imputation; CI = confidence interval.

## Discussion

This is the first study to explore associations between CYP’s beliefs about pain (or their “personal models” of pain) in JIA with their levels of pain and disease activity. This cross-sectional, retrospective analysis of a well-characterized sub-cohort of CYP with JIA assessed at a median of 5 years postdiagnosis found that CYP with JIA and high levels of pain, regardless of disease activity levels, are more likely to perceive their pain as more threatening and less controllable compared with those who experience low levels of pain and disease. Furthermore, CYP who experienced both high pain and high disease are also likely to have more responsive and anticipatory emotional representations compared to those with low levels of pain and disease. The differences were found across those with different pain levels (e.g., no difference was observed in participants with low pain within both the low disease and high disease activity categories). These findings contribute further evidence for the important role of pain beliefs, particularly when it comes to chronic pain conditions.

While it has been recognized that there is a subset of CYP who experience pain regardless of disease activity and treatment (Bromberg et al., 2014), this is the first study to compare the differences in those CYP who have low pain/low disease activity with those CYP who have high pain/low disease activity. A recent research priority setting in the United Kingdom identified that allied health professionals wanted to know how best to recognize children who are at risk of persistent pain (Smith et al., 2022), and this study begins to address this question. It was already known that CYP who experience higher pain intensity are at higher risk for persistent pain (Rashid et al., 2018) and negative long-term disease outcomes (Arnstad et al., 2019). This study has now found that CYP with high levels of pain also perceive a higher threat to their daily life regardless of disease activity.

Threat in this study was defined and assessed through a combination of beliefs about consequences—the impact of pain on their lives and activities, perceived chronicity of pain, perceived recurrence of pain, and perceived predictability of pain—if they are able to perceive cycles or patterns in their pain/flare-ups (Hagger & Orbell, 2022). This perception of threat was strongly related to higher levels of representations of responsive and anticipatory emotions. Previous studies highlighted that CYP with JIA who had more negative emotional representations (e.g., worry, frustration) perceived pain as a threat to their social identity and being seen as “normal” in comparison to their peers and tended to focus on managing the pain rather than trying to maintain or redefine normality (Ghio et al., 2022).

The theoretical model underpinning the current study has been applied to adult cohorts with chronic pain in order to reduce threat of pain, address reactive and anticipatory emotional representations to increase perceived control over pain (both self-efficacy and treatment), and reduce fear (Bunzli et al., 2017; Caneiro et al., 2022; O’Sullivan et al., 2018). The current study provides evidence that this approach could potentially be applicable to pediatric populations, calling for further work on how these working “personal models” can impact pain assessment, management, and communication in chronic inflammatory conditions, such as JIA.

Research has established that pain trajectories are not reliant on disease activity (e.g., active joint count) but has shown (and tested in animal models) those who had more swollen and limited joints (seen as evidence of prior disease activity) had worse pain but only in those with higher sensitivity (Learoyd et al., 2019). Increased disease duration is thought to play an important role in persistence of pain (Learoyd et al., 2019; Tollisen et al., 2018). However, this was not consistent with the demographic information in the current work that investigated data from an inception cohort design study. Those who were in the high pain/high disease group were slightly older at diagnosis and had fewer years since diagnosis. Although recent evidence has shown that adolescents experience greater delays to treatment in JIA, they may therefore have longer to treatment than as shown in the data (Shoop-Worrall et al., 2022).

The current study suggests that we need mechanisms in place to identify those CYP who will continue to experience pain. Therefore, pain should be proactively assessed and managed from diagnosis of JIA. This approach could prevent chronicity even if it is considered secondary pain to the underlying musculoskeletal disease. Findings suggest that there may be a cumulative effect of both high pain and high disease activity, impacting how CYP perceive their inflammatory condition and related chronic musculoskeletal pain. This fits with previously published findings highlighting that pain was the main determinant of well-being but has a bigger impact on those with greater disease activity (Alongi et al., 2019) with similar burden to those people with multimorbidity of two or more long-term conditions (Skou et al., 2022).

### Limitations

The findings need to be considered within the context of some limitations, as this is a UK study and may not be reflective of other populations or experiences in other healthcare systems. Furthermore, these findings demonstrate an association and not causality. The pain perception questionnaire was not introduced at the inception of the study; therefore, some participants did not complete it at diagnosis, and this is why there was an average of 5-year disease duration. This duration was significantly different across the groups and may account for low disease activity in some groups due to the time for treatment and management to be effective. However, the duration allowed us to examine CYP’s “personal model” of pain for those who continue to have high levels of pain regardless of disease activity. While the grouping of the disease activity allowed for a composition of active and limited joints and grouped similar PGAs, JIA is a heterogenous disease in terms of symptoms and signs over time (Shoop-Worrall et al., 2021), and more research is needed to investigate the groups identified in this study (e.g., low pain and high disease—had a large representation but was not significantly different from the low pain, low disease group). Although this study did not find a significant difference between ILAR classifications, this is in contrast with studies examining trajectories over time which have found differences in these groups. It is likely that, with a larger sample size, these differences may have also been found between the ILAR classifications. However, with observational research, there is an inherent *inclusion bias* that will evaluate data from centers that recruit for the cohort study, and then those who would have consented to take part in the research and completed the pain questionnaire. The proportions in each grouping do follow similar patterns to previous research into trajectories, for example, in the United Kingdom (Shoop-Worrall et al., 2021) and in cohorts outside of the United Kingdom (Shiff et al., 2018, 2023). Together, this suggests that the groupings are representative of groups present in clinic. A greater sample size, in particular of those with high pain/low disease, would be valuable to understand if beliefs around pain control, emotional representation, and perceived threat of those in this study are reflective of those in the wider JIA population.

Furthermore, the cJADAS-10 is a composite score of variables that indicate criteria for improvement and are not intended as an objective measure of active disease. As such, it relies on perception of well-being, which may have been influenced by the healthcare professional’s perception of pain. Research has shown inconsistency among scores such as the PGA that are not reliable (Backström et al., 2023). The score may be representative of different aspects of the disease beyond active and limited joints (e.g., research has found that even without active joint count the score is not always scored zero) with pain presentation being a reason given (Alongi et al., 2022). This suggests there might be more complexity in these constructs and how healthcare professionals, CYP, and parents/carers perceive disease and pain.

### Clinical implications

The current findings pertaining to a working “personal model” of pain for CYP with JIA (that includes beliefs of threat, control, and emotional representation) map onto areas that can be addressed through pain neuroscience education that has been found to successfully lead to reconceptualization of pain, decrease pain and disability, reduce fear of movement, and lower risk of pain “catastrophising” (Cuenca-Martínez et al., 2023). The protective function of pain is less apparent in this context when disease is low, and there is a need for restructuring in how we acknowledge pain beliefs and address them in clinic (Lewis & O’Sullivan, 2018). These results also pinpoint toward potential targets for future research into pain education interventions that might reduce the risk for pain chronification regardless of disease activity. The findings highlight the importance of enabling both clinicians and CYP with JIA to understand the role played by specific psychological factors and how they might contribute to pain experiences and mechanisms such as central sensitization (Ghio & Peters, 2024).

## Conclusion

The study adds to the evidence by demonstrating that disease activity alone is not a clear indicator of likely outcomes. It reveals that key beliefs about pain which, when viewed together, provide a “working” personal model of individual experiences of pain, are important contributing factors to the experience of pain. Perceptions of pain are important to consider from diagnosis, although more work is required to examine pain perceptions from the perspective of healthcare professionals and parents/carers. The findings demonstrated that those with the highest level of pain severity, regardless of their disease activity, perceived pain as more of a threat, having higher responsive and anticipatory emotions and feeling less control over their pain.

## Supplementary Material

jsaf024_Supplementary_Data

## Data Availability

Information regarding applying for access to CAPS data can be found online.
