# Supplementary material for: The interplay between pain and disease activity: personal models of pain beliefs and emotional representations in children and young people with juvenile idiopathic arthritis in a UK nationwide prospective inception cohort
Source: J Pediatr Psychol. 2025 Apr 23;50(10):937–45. doi: 10.1093/jpepsy/jsaf024 (PMC12531478; doi:10.1093/jpepsy/jsaf024)
Supplement: jsaf024_Supplementary_Data [file jsaf024_supplementary_data.docx]

**Power Calculation**

To test for univariable similarity across all four groups, three with sample size=50, and one with sample size=15, with mean pain control difference values of at least 15 (tested average +/- 15), within group variance of 500 and significance set at 0.05, the analysis has at least 95% power.

**Supplementary Table:** Differences in characteristics between those included and excluded from the current study

| **Characteristic at diagnosis** | **N(%) or median (IQR)** | | **p-value** |
| --- | --- | --- | --- |
|  | ***Included in study*** | ***Excluded from study*** |  |
| N | 363 | 1402 | - |
| Age at diagnosis (yrs) | 8.1 (4.6, 12.0) | 7.7 (3.4, 12.0) | 0.200 |
| Symptom duration to diagnosis (months) | 4.4 (2.2, 7.7) | 4.5 (2.1, 8.4) | 0.635 |
| Female | 188 (68.1) | 740 (64.0) | 0.153 |
| White ethnicity | 282 (87.4) | 1018 (84.1) | 0.116 |
| ILAR category | | | 0.002 |
| Systemic JIA | 20 (5.8) | 88 (6.7) |  |
| Oligoarthritis | 140 (40.8) | 673 (50.9) |  |
| RF- polyarthritis | 95 (27.7) | 271 (20.5) |  |
| RF+ polyarthritis | 17 (5.0) | 46 (3.5) |  |
| Enthesitis-related JIA | 23 (6.7) | 70 (5.3) |  |
| Psoriatic JIA | 28 (8.2) | 86 (6.5) |  |
| Undifferentiated JIA | 20 (5.8) | 88 (6.7) |  |
| Active joint count | 2 (1, 6) | 2 (1, 5) | 0.051 |
| Limited joint count | 1.5 (0, 4) | 1 (1, 3) | 0.401 |
| Physician’s global assessment (cm) | 2.9 (1.6, 5.0) | 2.9 (1.5, 5.1) | 0.846 |
| Parental global evaluation (cm) | 2.1 (0.5, 5.0) | 2.4 (0.6, 5.1) | 0.326 |
| CHAQ | 0.6 (0.1, 1.4) | 0.8 (0.3, 1.5) | 0.020 |
| ESR (mm/hr) | 20 (8, 49) | 24 (9, 52) | 0.189 |
| Pain (cm) | 2.6 (0.5, 5.6) | 3.0 (0.8, 6.0) | 0.247 |
